# Uncertainties in Markov State Models of Small Proteins

**DOI:** 10.1021/acs.jctc.3c00372

**Published:** 2023-08-04

**Authors:** Nicolai Kozlowski, Helmut Grubmüller

**Affiliations:** Department of Theoretical and Computational Biophysics, Max-Planck-Institute for Multidisciplinary Sciences, Göttingen 37077, Germany

## Abstract

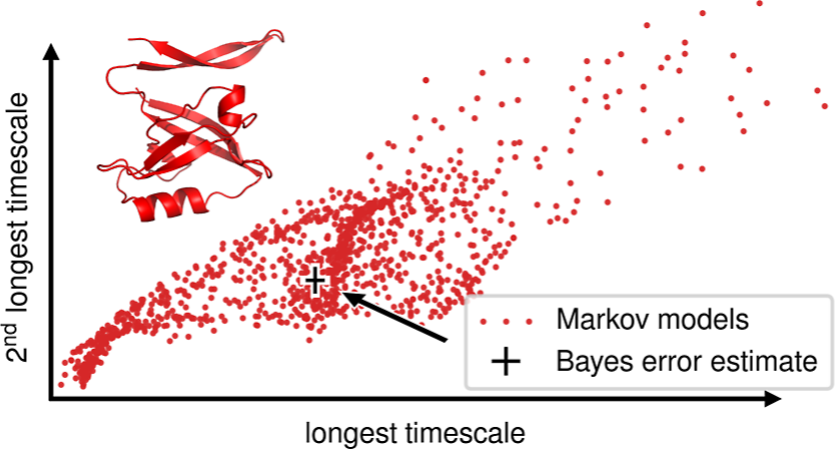

Markov state models are widely used to describe and analyze protein dynamics based on molecular dynamics simulations, specifically to extract functionally relevant characteristic time scales and motions. Particularly for larger biomolecules such as proteins, however, insufficient sampling is a notorious concern and often the source of large uncertainties that are difficult to quantify. Furthermore, there are several other sources of uncertainty, such as choice of the number of Markov states and lag time, choice and parameters of dimension reduction preprocessing step, and uncertainty due to the limited number of observed transitions; the latter is often estimated via a Bayesian approach. Here, we quantified and ranked all of these uncertainties for four small globular test proteins. We found that the largest uncertainty is due to insufficient sampling and initially increases with the total trajectory length *T* up to a critical tipping point, after which it decreases as , thus providing guidelines for how much sampling is required for given accuracy. We also found that single long trajectories yielded better sampling accuracy than many shorter trajectories starting from the same structure. In comparison, the remaining sources of the above uncertainties are generally smaller by a factor of about 5, rendering them less of a concern but certainly not negligible. Importantly, the Bayes uncertainty, commonly used as the only uncertainty estimate, captures only a relatively small part of the true uncertainty, which is thus often drastically underestimated.

## Introduction

Markov state models (MSMs) are widely used as coarse-grained models of the conformational dynamics of biological macromolecules such as proteins.^1–6^ They rest on a partitioning of the configurational space of a fully atomistic description of the molecular dynamics (MD) into discrete states and transition rates between these states.^1,2^ Often, but not necessarily, these states describe conformations or conformational substates.^7^ Typically, MSMs are constructed from atomistic MD simulations.^8–10^ Their main purpose is to provide an analytic description and to access long time scales otherwise not accessible to unbiased MD simulations, e.g., to unravel kinetic pathways and to estimate characteristic time scales of protein folding,^11–14^ of ligand binding,^15,16^ and of self-assembly.^17^

However, to compute sufficiently long (or sufficiently many) unbiased MD trajectories for MSMs of proteins to converge is quite challenging despite impressive computational and algorithmic advances^18^ as well as increasingly efficient sampling protocols.^19–23^ Obviously, conformations that belong to the equilibrium ensemble but are not visited by the MD trajectories will also be missing in the derived Markov model. However, even for those conformations that are visited, the number of transitions between these may be small, and hence, transition rate estimates may be inaccurate. These shortcomings—collectively often referred to as insufficient sampling or sampling problem—are generally assumed to be the main sources of MSM uncertainties.^24,25^

Bayesian approaches serve to quantify part of the sampling uncertainties. To this end, sample MSMs are drawn from the Bayesian posterior probability distribution *p*(**P**|**C**) using Markov chain Monte Carlo (MCMC) methods.^26,27^ Here, **C** is the count matrix for all transitions between Markov states observed in the MD trajectories, and **P** is the MSM transition probability matrix. However, this posterior distribution only captures uncertainties due to small transition counts between known Markov states but misses uncertainties due to other sources. Unseen conformations, discretization errors, memory effects, or the impact of parameter choices during MSM construction steps such as dimension reduction or transition counting all induce uncertainties that are not captured by the Bayesian approach.

How large are these uncertainties and, consequently, how much do Bayesian error estimates underestimate the true error under realistic simulation conditions and for typical simulation systems? Recently, Noé et al. have addressed uncertainties arising from the limited number of counted transitions^27^ and, in particular, correlations between these transitions and suggested methods to obtain uncorrelated transition counts **C** from discrete trajectories to enhance the accuracy of the Bayesian uncertainty estimates.^28^ However, other uncertainties, which are not captured by the Bayesian posterior distribution, typically also contribute to the total uncertainty but have, to our best knowledge, so far not been systematically quantified.

Here, we address these questions empirically by estimating these additional MSM uncertainties for several typical proteins using an extensive series of increasing sample sizes, the number of used trajectories, and cross-validations. To this aim, we first identified a total of seven sources of uncertainty within state-of-the-art and widely used MSM construction pipelines.^6,29^ The first is due to missed conformations; four more considered types of uncertainties are induced by somewhat arbitrary parameter choices, such as MSM lag time τ_MSM_, number of Markov states *k*, tICA lag time τ_tICA_, and dimension reduction variance cutoff *v*_c_. We further considered the uncertainty induced by the stochastic element of the construction pipeline inherent to *k*-means clustering and finally the statistical uncertainty arising from the limited number of transitions between conformational states observed in the trajectory. The latter is often assumed to be the dominant source of uncertainty and usually quantified by the Bayesian posterior *p*(**P**|**C**)^13,16,27^ or bootstrapping techniques.^14,15,30^ To quantify these uncertainties, we focused on the longest relaxation time scales of the MSMs as these often describe biologically relevant processes but also because these are assumed most sensitive to the above sources of uncertainty.

As prototypic examples and test systems, we chose four small macromolecules, (1) the human Pin1 WW domain (Pin WW), a small protein module with 35 residues (PDB: 2F21),^31^ (2) the homeodomain of mouse hepatocyte nuclear factor 6 (HNF HD) with 50 residues (PDB: 1S7E),^32^ (3) the XPC-binding domain of protein hHR23B (XPCB) with 72 residues (PDB: 1PVE),^33^ and (4) the protein neuronal nitric oxide synthase (nNOS) with 112 residues (PDB: 1QAU).^34^ These proteins have in common the fact that they are single domain, have no prosthetic groups, and cover different topologies as well as the size range of typical small globular proteins. They are also chosen to be small enough that our extensive sampling can be assumed to provide accurate uncertainty estimates.

## Methods

### MD Simulations

For the main analysis of this study, we performed 288 1 μs long MD simulations each for Pin WW, HNF HD, XPBC, and nNOS. Additionally, five 32 μs long MD simulations were performed for Pin WW to compare sampling strategies.

All simulations were performed using the Gromacs 2018 software package^35^ with the Amber99sb-ildn force field^36^ and TIP4P-Ew water model.^37^ Starting structures for all simulations were taken from the PDB,^38^ entries 2F21,^31^ 1S7E,^32^ 1PVE,^33^ and 1QAU^34^ and placed in a triclinic simulation box which is larger than the protein by 1.5 nm in all directions. Solvent and ions (Na^+^ and Cl^–^) were added, establishing a salt concentration of 0.15 mol L^–1^ and neutralizing the overall system charge. Energy minimization was performed using GROMACS steepest descent until convergence to machine precision (single precision, ≤5 × 10^4^ steps). Subsequently two equilibration runs were performed for 0.5 (*NVT*) and 1 ns (*NPT*) at an integration time step of 2 fs, followed by the actual production runs with a length of 1 μs (*NPT*, 32 μs for the long simulations) at an integration time step of 4 fs using virtual sites. A velocity rescaling thermostat^39^ was applied at *T* = 300 K with temperature coupling constant τ_T_ = 0.1 ps. Isotropic pressure coupling was applied at *p* = 1 bar with pressure coupling constant τ_p_ = 1 ps using Berendsen pressure coupling^40^ during equilibration and Parrinello–Rahman pressure coupling^41^ in the production run. All bond lengths were constrained using the Settle algorithm^42^ for the solvent and Lincs(43) for the solute, with a Lincs order of 4 during energy minimization and equilibration and a Lincs order of 6 in the production run. Van der Waals forces were cut off at 1 nm. Coulomb forces were calculated using the particle mesh Ewald method (PME)^44^ with a real-space cutoff of 1 nm, a PME order of 4, and a Fourier grid spacing of 1.2 Å. In the production runs, coordinates were recorded every 10 ps.

### MSM Construction

All MSMs were constructed from these MD trajectories in several processing steps as follows using Pyemma 2 software.^10^ Only the Cartesian coordinates of C_α_-atoms were considered.

First, we computed a time-lagged independent component analysis (tICA)^45,46^ to project the trajectory onto the slowest collective motions, which has been shown to provide dimension reductions that result in particularly accurate MSMs^46,47^ and for which criteria for insufficient sampling have recently been obtained.^48^ After projecting, the tICs were rescaled with their respective eigenvalues, such that Euclidean distances in the projection correspond to kinetic distances.^49^ The two required parameters, lag time τ_tICA_ and variance cutoff *v*_c_, were both used in our systematic parameter scan.

Second, *k*-means++ clustering^50,51^ was performed on the obtained tICA projections, which has been shown to be among the best choices of clustering algorithms for constructing MSMs from MD trajectories^52^ and is widely used.^10,11,25,53–55^

Third, transition counts were estimated from the discrete trajectories using a sliding window counting scheme with correction for statistical inefficiencies,^28^ as recommended in Pyemma 2 for use with Bayesian transition probability estimates.^10^ Transition counts *c*_*i*→*j*_(τ_MSM_) depend on the MSM lag time τ_MSM_ and form a count matrix **C** (τ_MSM_). The lag time τ_MSM_ was included within our systematic parameter scan.

Fourth, transition probabilities *p*_*i*→*j*_ were estimated using the Bayesian posterior probability distribution *p*(**P**|**C**) ∝ *p*(**C**|**P**)*p*(**P**) ∝  under the constraints of detailed balance.^27^ The sparse prior  was chosen to enforce **P** to have the same sparsity pattern as **C**,^27^ i.e., transitions that where observed neither in forward nor in backward direction have zero probability. Using MCMC sampling, 100 sample MSMs were drawn from this posterior distribution to obtain Bayes uncertainty estimates.

MSMs were constructed for 10 separate trajectory sets for total lengths *T* = 1, 2, 4, 8, and 16 μs, 9 sets for *T* = 32 μs, and 4 sets for *T* = 64 μs, each with all combinations of the parameters listed in Table 1. For single trajectory sampling, five different trajectories were used for all *T* = 1, 2, 4, 8, 16, and 32 μs. Every MSM construction was repeated five times with different random seeds.

**Table 1:** MSM Parameters

molecule	tICA lag τ_tICA_ [ns]	variance cutoff *v*_c_	number of Markov states *k*	MSM lag τ_MSM_ [ns]
Pin WW	0.15, 0.21, 0.27, 0.33	0.85, 0.90, 0.95, 0.99	1500, 2200, 2800, 3500	15, 22, 28, 35
HNF HD	0.5, 1.5, 2.5	0.50, 0.65, 0.80	100, 250, 400	1.0, 2.5, 4.0
Fas 1	1.0, 2.5, 4.0	0.5, 0.7, 0.9	250, 500, 750	3.5, 5.0, 6.5
XPCB	0.5, 1.5, 2.5	0.50, 0.65, 0.80	100, 250, 400	1.0, 2.5, 4.0

Our particular choices for τ_tICA_, *v*_c_, *k*, and τ_MSM_ were based on three criteria. First, the longest time scale of MSMs constructed from single 1 μs trajectories does not show a strong correlation with the parameter. Second, MSM estimation does not repeatedly crash within Pyemma due to the occupancy of Markov states approaching zero. Third, the longest time scales of the 100 sample MSMs resemble a log–normal distribution in nearly all cases.

### Uncertainty Estimates

The uncertainty of the MSMs was estimated using a hierarchical scheme as follows. First, the Bayesian uncertainty estimate σ_B_ = σ(*t*′) was calculated from the 100 sample MSMs as the standard deviation of their longest logarithmic time scales *t*′ = log_10_*t* = log_10_(−τ_MSM_/ln λ).^29^ For all other uncertainty estimates, the 100 sample MSMs were represented by their mean longest logarithmic time scales *t*_B_ = ⟨*t*′⟩. In some cases, the 100 *t*′ distribution was heavy-tailed, reaching time scales >10^4^ s. In these cases, the MCMC sampling was considered to not be converged and therefore excluded from analysis. These cases were detected using a Shapiro–Wilk normality test^56^ on the longest and second longest logarithmic time scales with *W*_crit_ = 0.95.

Second, to estimate the uncertainty σ_RS_ due to the stochastic element of *k*-means, five MSMs were constructed with identical parameters and input trajectories but different random seeds. The uncertainty was calculated as the weighted standard deviation  of the five *t*_B_. For all subsequent uncertainty estimates, the five MSMs were represented by their weighted mean
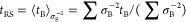


Third, uncertainties due to limited sampling, parameter choice, and total uncertainty were estimated. Sampling uncertainty σ_sampling_ was estimated as the weighted standard deviation  of MSMs constructed from different trajectory sets of equal *T* with identical parameters. The uncertainty of choosing a particular parameter, e.g., *k*, was estimated as the weighted standard deviation  of MSMs constructed with different *k* but identical other parameters and from the same trajectory set. The total uncertainty was estimated as the weighted standard deviation  of all MSMs for given *T*, including all different parameter choices and input trajectories. It does not correspond to the sum of the other uncertainties. The latter estimate is likely the most accurate; however, because only one estimate for each *T* and for each protein is obtained, no confidence interval can be provided.

## Results and Discussion

### Comparing Sources of Uncertainty

For each of the four test proteins Pin WW, HNF HD, XPBC, and nNOS, we generated 288 trajectories of length 1 μs each. From these, a total of 104,789 MSMs were constructed using different amounts of sampling and different choices for the construction parameters mentioned in the Introduction. All uncertainties were estimated from these MSMs as described in the Methods section. Briefly, Bayes uncertainties were estimated from standard deviations of logarithmic time scales of a sample of MSMs drawn from the posterior probability distribution. Similarly, random-seed uncertainties were calculated from five repeats of the MSM construction with different random seeds. Parameter-choice uncertainties were estimated from MSMs which differ in the particular parameter but share all other parameters and their respective input trajectories. Similarly, sampling uncertainties were calculated from MSMs constructed from different trajectories but sharing all parameters. Total uncertainties were estimated from all available MSMs and thus are not simply the (squared) sum of the above uncertainty contributions.

**Figure 1:**
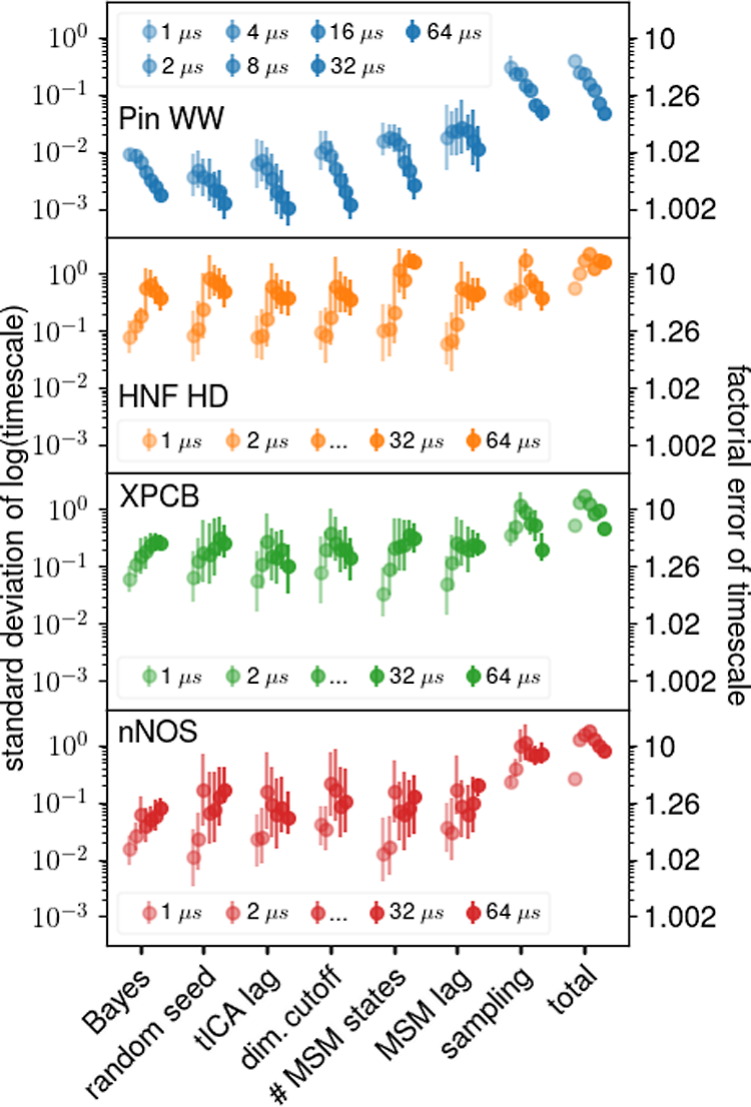
Comparison of uncertainties in the longest relaxation times from different sources for four different proteins (colors). Mean uncertainties are denoted by circles. The error bars represent the central 66% of the observed distributions, not the error of the mean. Total trajectory lengths used to construct the MSMs are indicated by color saturation and are slightly horizontally displaced for clarity. The standard deviation of logarithmic time scales translates to a factor describing the time scale uncertainty in linear space, e.g., a factor of 2 means that 68% of the time scales lie within twice and half the given value. This factor is shown on the right *y*-axis.

Figure 1 shows the obtained estimates for uncertainties of the longest time scales. The error bars indicate the 66% confidence interval of the above distributions. As can be seen, in most cases—but not all—the source of the largest uncertainty is limited sampling. The total uncertainty is within the error bars of the sampling uncertainty in 17 out of a total of 28 cases (≈61%). In contrast, the Bayesian estimate significantly underestimates the total uncertainty. There are three exceptions where the choice of the number of Markov states caused the largest uncertainty (HNF HD at total trajectory lengths of 32 and 64 μs and XPCB at 64 μs). This unexpected result will receive closer attention further below.

Overall, these results suggest that in most cases, limited sampling contributes most to the total uncertainty and should generally provide good uncertainty estimates. However, because many different sets of input trajectories are required, this estimate is generally computationally expensive and still tends to underestimate the true uncertainty. For this reason, the Bayesian estimate is frequently used as a substitute. One of the main findings of this study is that, unfortunately, the Bayesian estimate turns out to be one of the smallest contributions to the total uncertainty and thus tends to drastically underestimate the true uncertainty. In fact, the uncertainties of most other sources, e.g., the choice of the number of Markov states, are generally similar or even larger and will therefore be studied subsequently in more detail.

**Figure 2:**
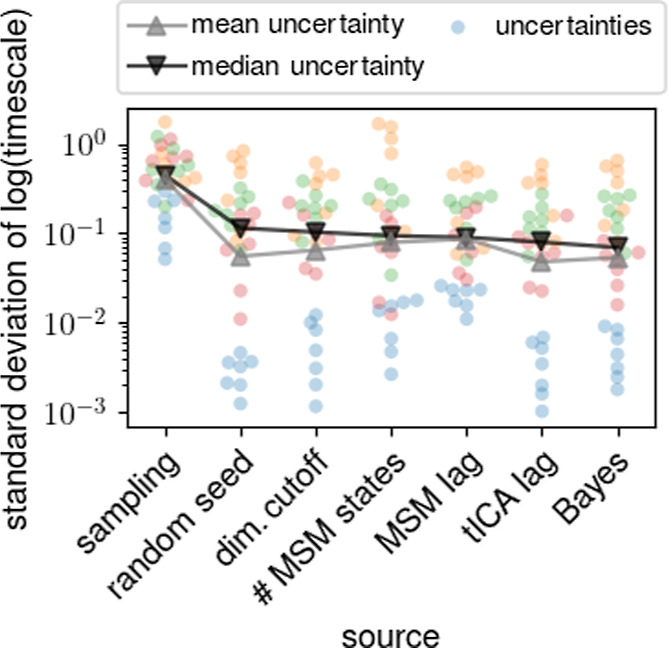
Ranking of uncertainties for the longest relaxation times from different sources. Blue, orange, green, and red circles denote uncertainties for Pin WW, HNF HD, XPCB, and nNOS, respectively, at different total trajectory lengths and the lines indicate mean (gray) and median (black) uncertainties. Small horizontal displacements are for visualization purposes only.

Before analyzing the individual sources of uncertainty in detail, Figure 2 shows the mean and the median uncertainty (gray and black curves, respectively) of each source for all four test proteins (colors) and for different total trajectory lengths. As can be seen, the average sampling uncertainty dominates and is larger than all other contributions by a factor of about 5. The median uncertainties follow a similar trend. In both cases, the uncertainties due to the choice of tICA lag time and the Bayes estimate tend to be the smallest. In particular, we were surprised to see that the inevitable uncertainty due to choice of random seed for the *k*-means clustering is similar to or larger than the widely used Bayes estimate.

### Uncertainty Scaling with Total Trajectory Length

**Figure 3:**
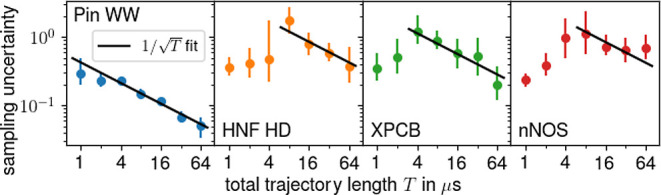
Change of sampling uncertainty with total trajectory length *T*. Mean uncertainties are denoted by colored dots with error bars representing 66% of the observed distributions. The solid black lines are -fits to the uncertainties at and beyond their respective maxima.

Having established that limited sampling is the source of the largest uncertainty, Figure 3 shows, for each protein, how this uncertainty changes with the total trajectory length *T*. As can be seen, for sufficiently large *T*, the uncertainty decreases  (black lines) for each protein, whereas for smaller *T*, the uncertainty increases with *T*. For Pin WW, the decrease already starts at *T* < 1 μs (data not shown), whereas for the larger proteins (to the right), this tipping point is between 4 and 8 μs. This trend supports the expectation that larger proteins require more sampling and also shows that the  behavior that is expected for sufficient sampling is indeed reached for all four test proteins. Larger or more conformationally diverse proteins, therefore, are likely out of reach even for massive sampling.

We attribute the above scaling behavior to a tug-of-war between two contributions to the sampling uncertainty, unexplored states, and low counts for transitions between explored states. For small *T*, the protein has explored only a small fraction of all states and, importantly, has explored different states in different trajectory sets. Therefore, the uncertainty due to unexplored states dominates. For increasing *T*, more different sets of states are explored within different trajectory sets, and thus, the sampling uncertainty increases. Eventually, most conformations are explored within each trajectory set such that the rate of increase with *T* becomes smaller. At the tipping point, the uncertainty due to the number of transition counts, which decreases , starts to dominate.

To test this scenario, we analyzed tICA projections of 1 μs trajectories. At this trajectory length, one would expect that for Pin WW, the majority of states is already explored, in contrast to the other three proteins. Indeed, projections of each Pin WW trajectory onto a common tICA subspace cover nearly identical regions, whereas much less overlap is seen for the other three proteins (data in Supporting Information).

### Comparing Sampling Strategies

**Figure 4:**
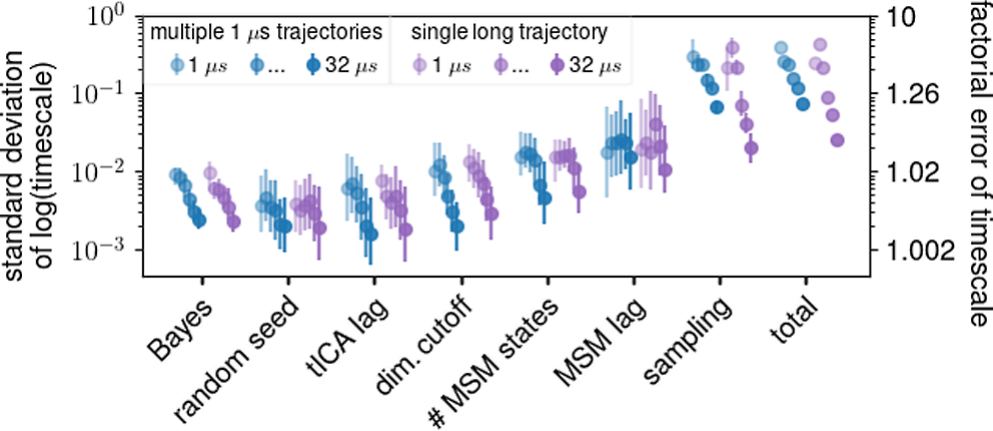
Comparison of uncertainties in the longest relaxation times of Pin WW for two different sampling strategies (blue and purple). For explanation of error bars, slight horizontal displacements, and right *y*-axis, see Figure 1.

So far, we have focused on one particular sampling strategy. Specifically, we increased the total trajectory length *T* by spawning additional 1 μs trajectories, all starting from the same structure. Using Pin WW as an example, we next investigated how different sampling strategies affect the individual uncertainties. To this end, Figure 4 compares this sampling strategy (blue, same data as shown in Figure 1) with a different one (purple) for which sampling is increased by prolonging a single trajectory. Due to the large computational effort involved, we restricted this analysis to *T* ≤ 32 μs. Overall, most uncertainties are rather similar for the two sampling strategies. The largest differences are seen for the sampling uncertainty. First, markedly higher accuracy is achieved for *T* ≥ 8 μs by single-trajectory sampling as compared to multitrajectory sampling (e.g., differing by more than a factor of 3 for *T* = 32 μs); second, the tipping point is shifted to larger *T* = 2 μs for single-trajectory sampling.

We attribute these differences to the fact that, for multitrajectory sampling, states close to the common starting structure are visited more often than those visited later. Accordingly, only a few transitions between the latter are observed, which therefore dominate the sampling uncertainty. This imbalance is much less pronounced for single-trajectory sampling and decreases with increasing trajectory length, thus explaining why the sampling uncertainty decreases faster with increasing *T* for the single-trajectory sampling.

This imbalance also explains the above shift in the tipping point. In particular, significant overlaps between different trajectory sets occur at shorter *T* for multitrajectory sampling than that for single-trajectory sampling. Therefore, for single-trajectory sampling, the tug-of-war between unexplored states and low transition counts is dominated by unexplored states for longer *T*.

### Systematic Error in Absolute Time Scales

**Figure 5:**
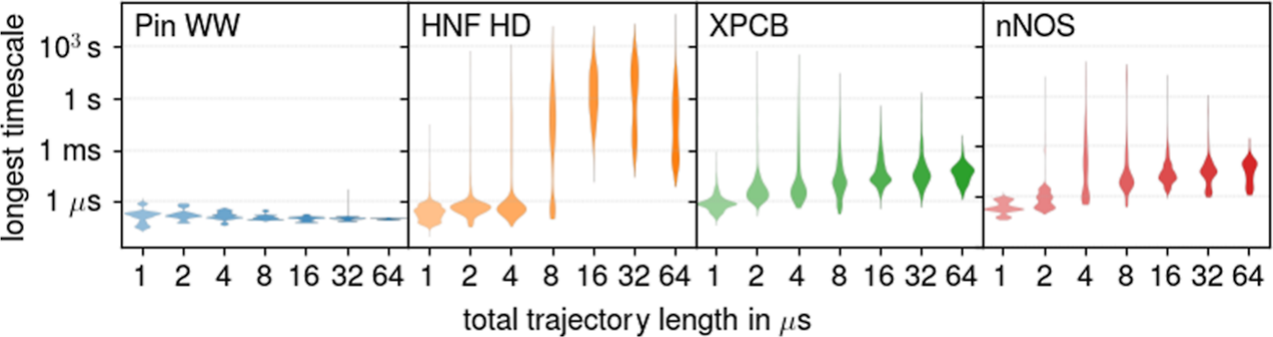
Distributions of longest relaxation times over all MSMs for the four considered proteins (colors), including different input trajectory sets and different construction parameters.

So far, we only reported on statistical uncertainties; next we will discuss systematic contributions to the total uncertainty. To this end, Figure 5 shows how the total trajectory length *T* affects the longest MSM time scales. As can be seen for the proteins HNF HD, XPCB, and nNOS, estimated time scales can be drastically longer for longer *T* as compared to those for short *T*. For Pin WW, in contrast, similar time scales are obtained for all *T* ∈ 1–64 μs. Also, for XPCB and nNOS, time scales increase gradually with *T* by almost 3 orders of magnitude. In contrast, for HNF HD time scales, a sudden jump is observed at *T* = 8 μs, by even 5 orders of magnitude.

The gradual increase can be explained in terms of new conformations being explored with increasing *T*, which therefore tend to be connected by increasingly slower transitions (data not shown). Indeed, no new conformations are explored for Pin WW and, accordingly, no increase of time scales is observed.

The occasional jump of the longest time scale, as seen for HNF HD, can be explained in terms of a sudden increase of the number of Markov states in the largest reversibly connected subset. This sudden increase, in turn, is explained as follows. Whereas new conformations are gradually explored with increasing *T* already before the jump, for many of those, no backward transitions are observed, and hence these are not yet part of the reversibly connected subset. These would act as a sink in the MSM and are therefore discarded.^10^ For *T* ≥ 8 μs, backward transitions occur from sufficiently many of those states, such that they become part of the largest reversibly connected subset, hence suddenly increasing the longest time scale.

### Uncertainties Due to Parameter Choices

**Figure 6:**
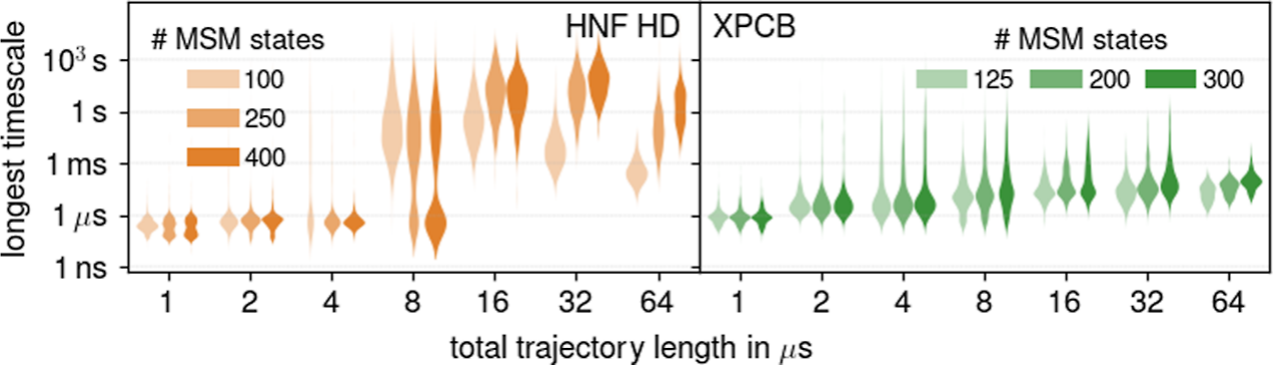
Correlation of absolute time scales with the number of Markov states *k* for HNF HD (orange) and XPCB (green) at different total trajectory lengths *T*. The violins indicate the distributions of longest relaxation time scales of all MSMs for given total trajectory length *T* and the number of Markov states *k* (indicated by color saturation).

Next, we will investigate the rare cases in which the source of the largest uncertainty is not limited sampling. In these cases, as shown in Figure 6, the choice of number of Markov states dominates the total uncertainty for HNF HD at *T* ≥ 32 μs and XPCB at *T* = 64 μs. As can be seen, here, the absolute time scales increase with the number of Markov states. The absolute time scales also increase with MSM lag time, albeit to lesser extent (data not shown).

Such a strong dependence is unphysical and can therefore serve as a warning flag that the obtained MSMs are problematic. Such dependence can be due to an inadequate parameter range for the number of Markov states or inadequate preprocessing parameters such as tICA lag time or dimension cutoff.^57^ In all other cases, where no or only weak correlations between parameters and the longest time scale were observed, limited sampling was the dominant source of uncertainty.

### Uncertainty in Shorter Time Scales

So far, we only considered the longest time scale of the MSM; next we will investigate the shorter (second to eighth-longest) time scales. These time scales describe subsequently faster equilibration processes for which sufficient sampling is reached at shorter *T*. We selected Pin WW as an example here for which we obtained the most comprehensive sampling.

**Figure 7:**
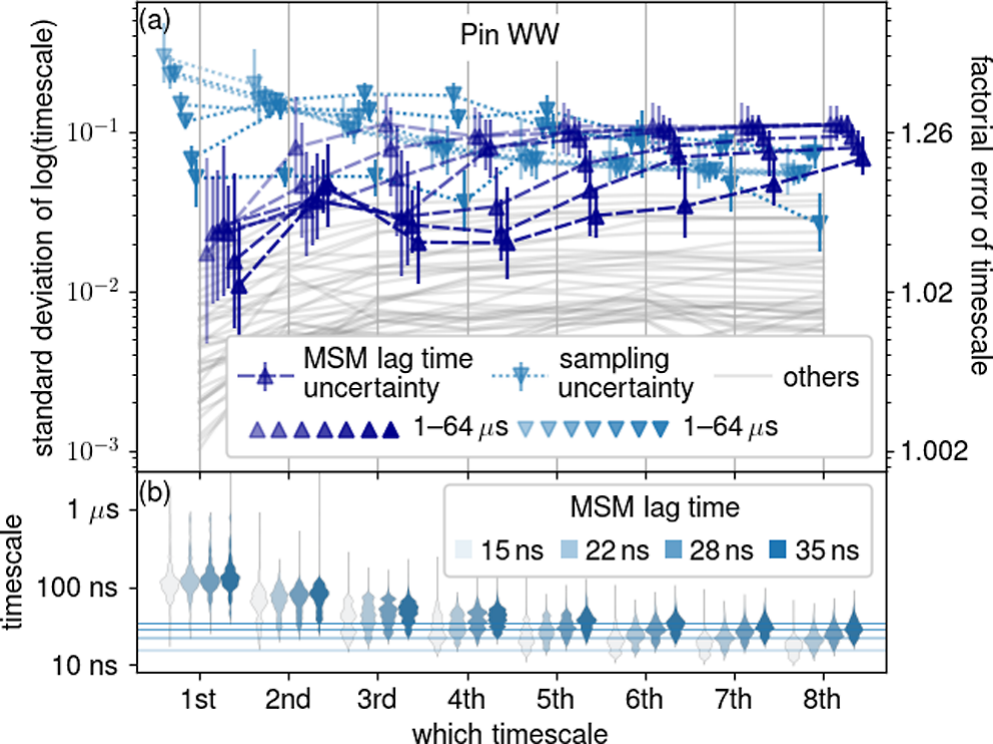
Faster time scales of Pin WW MSMs and their, respective, uncertainties. (a) Sampling uncertainty and MSM lag time uncertainty are denoted by light blue and purple triangles, respectively, for the longest to eighth-longest time scale. Other uncertainties are represented by gray lines. For explanation of error bars, slight horizontal displacements, and right *y*-axis, see Figure 1. (b) Violins depict the distributions of longest to eighth-longest absolute time scales. Here, higher-saturation violins correspond to MSMs with longer MSM lag times, and every violin contains MSMs of all total trajectory lengths *T* ∈ 1–64 μs. The four MSM lag times used are shown as horizontal lines.

As can be seen in Figure 7 (top)—and as expected from the increasingly faster relaxation—generally, the sampling uncertainty (light blue) decreases from the longest to the eighth-longest time scale. In contrast, the MSM lag time uncertainty (purple) increases, eventually surpassing the sampling uncertainty. Comparison with all other sources of uncertainty (gray) shows that limited sampling still remains the second-largest contribution. These trends are generally more pronounced for shorter total trajectory lengths *T*.

As can be seen in Figure 7 (bottom), the absolute time scales increase with MSM lag time. Again, such a dependence can serve as a warning flag of inadequate parameter choice. Here, the dependence is more pronounced for shorter time scales, whose absolute lengths are close to the MSM lag times (shown as horizontal lines in the lower part of Figure 7). Because the MSM lag time limits the time resolution of the model, this observation suggests that the faster processes described by the shorter time scales are not resolved by our MSMs.

Taken together, our results suggest that for appropriate choices of the MSM parameters, limited sampling is the dominant source of uncertainty. Furthermore, the dependence of a time scale on a particular parameter can flag possibly inaccurate MSMs.

## Conclusions

Using four typical globular proteins, we systematically quantified different contributions to the uncertainty in MSMs constructed from MD simulations with a particular focus on the largest relaxation times that are often relevant for biomolecular function. Our first main finding is that limited sampling is the dominant source of uncertainty. This contribution arises from both unseen conformations as well as low counts of conformational transitions. A noteworthy observation is that, although this uncertainty initially increases with the total trajectory length *T*, it eventually reaches a tipping point at a certain *T*_tp_ and then decreases. For the four small to midsize proteins studied here, the tipping point was indeed within the reach of extensive MD simulations, such that a ubiquitous decrease of  beyond *T*_tp_ was observed. This scaling behavior, when observed, can serve to estimate how much simulation time is required to reach a desired accuracy. For our four test proteins, the location of the tipping point *T*_tp_ is clearly determined by the size of the protein and by the complexity of its dynamics. Notably, limited sampling not only gives rise to large uncertainties but also can cause large systematic errors. For example, and particularly for small *T* ≤ *T*_tp_, time scales were often underestimated. For very long *T* ≫ *T*_tp_, we expect this systematic error to decrease with the uncertainty.

In a recent report, Suárez et al. performed a parameter grid search and cross-validation of MSMs based on their longest time scales (truncated VAMP-2 score) for model selection.^58^ Here, too, average deviations between training and test scores (resembling sampling uncertainty) appear to be larger than the deviations between training scores of MSMs with different parameters. Overall, although no quantitative analysis was performed, these results appear to be consistent with our present study.

Also recently, He et al. reported an MSM-based analysis of protein–protein association from coarse-grained simulations.^59^ They estimated binding free energies for different *T*, the errors of which appear to decrease . They also report that the choice of the number of retained tICA dimensions caused only small uncertainties in the equilibrium properties. Our results extend these findings to relaxation time scales of protein conformational dynamics from atomistic simulations.

It is worth noting that a single long trajectory yields better sampling and, hence, improved accuracy than multiple shorter trajectories of the same accumulated length started from the same structure. This effect is particularly pronounced for sampling the relevant long time scales. Of course, many advanced sampling techniques have been developed,^19,22,24,60–64^ which generally provide better sampling efficiency even for very complex biomolecules. Nevertheless, the above two simple sampling strategies are still widely used,^13,58,65–71^ and a more systematic comparison of these advanced methods is clearly required, although beyond the scope of this article.

Of course, because calculating many trajectories scales much better on parallel computers, it may still be preferable. Also, it is clearly preferable to start these from independent structures, which, however, are rarely available. Rather, the typical situation is that only one experimental structure is available such that generating largely independent structures would require additional computational effort. For a fair comparison, this effort would have to be taken into account, too.

A recent study by Bacci et al. reported that MSMs can generally recover correct equilibrium distributions from multiple short simulations all started from a small region in phase space,^72^ resembling multiple trajectories from a common starting structure. Our results additionally suggest that MSMs can also recover dominant relaxation time scales, i.e., kinetics, from such trajectories. Contrary to Bacci et al., we found that applying detailed-balance constraints does not compromise that ability (data not shown). We refrained from a more in-depth analysis, as it would further distract us from our main focus.

The second main result is that the widely used Bayesian estimate for MSM uncertainties captures only a small part of the total relaxation time uncertainty and therefore bears the risk of drastically overestimating the achieved accuracy. More realistic uncertainty estimates and validations are obtained using simulation series of increasing trajectory lengths or number. The price to pay is more computational cost as only a small part of the trajectories will serve the MSM construction. One might be tempted to construct an MSM on all available trajectories for which, however, no reliable error estimate would be obtained. Furthermore, this MSM is not guaranteed to be more accurate.

Other contributions to MSM uncertainties can arise from the particular parameter choice in a conventional MSM construction pipeline, especially the number of Markov states, the amount of dimension reduction, and the MSM lag time. We observed that the uncertainties due to each of these parameters show a plateau within an appropriate parameter range, in which case their contribution to the total uncertainty is generally small, albeit not negligible. It follows that large variations of MSM relaxation times with one of these parameters can flag inaccurate MSM.

Of course, other sources of MSM uncertainties exist that have not been discussed here. The most obvious example is the choice of different elements of the MSM construction pipeline, for which here we have chosen the widely used methods implemented in Pyemma 2^10^ to exclusively construct microstate models. One could, for example, choose a different clustering algorithm (Ward’s method, *k*-centers, ...),^52^ or exchange (parts of) the construction pipeline with a neural network,^73,74^ or additionally coarse-grain the MSM (using PCCA^75^) to obtain more comprehensible macrostate models at the cost of accuracy.^29^ Also, the underlying MD simulation method suffers from inaccuracies, e.g., due to the choice of force field or starting structure. Quantifying the effect of these clearly deserves future study.

## Supporting Information Available

TICA projections of 64 1 μs MD trajectories for each protein, highlighting areas covered by individual trajectories (PDF)
