# Supplementary material for: Uncertainties in Markov State Models of Small Proteins
Source: J Chem Theory Comput. 2023 Aug 4;19(16):5516–24. doi: 10.1021/acs.jctc.3c00372 (PMC10448719; doi:10.1021/acs.jctc.3c00372)
Supplement: Supplementary file 1 — ct3c00372_si_001.pdf [file ct3c00372_si_001.pdf]

Supplementary Material for: Uncertainties in Markov State Models of small Proteins

Nicolai Kozlowski* and Helmut Grubmüller*

Department of Theoretical and Computational Biophysics, Max-Planck-Institute for Multidisciplinary Sciences, Göttingen, 37077, Germany

E-mail: nkozlow@mpinat.mpg.de; hgrubmu@mpinat.mpg.de

As can be seen by comparing Fig. S1 with Figs. S2-S4, projections of each Pin WW trajectory onto a common tICA subspace cover nearly identical regions, whereas much less overlap is seen for the other three proteins.

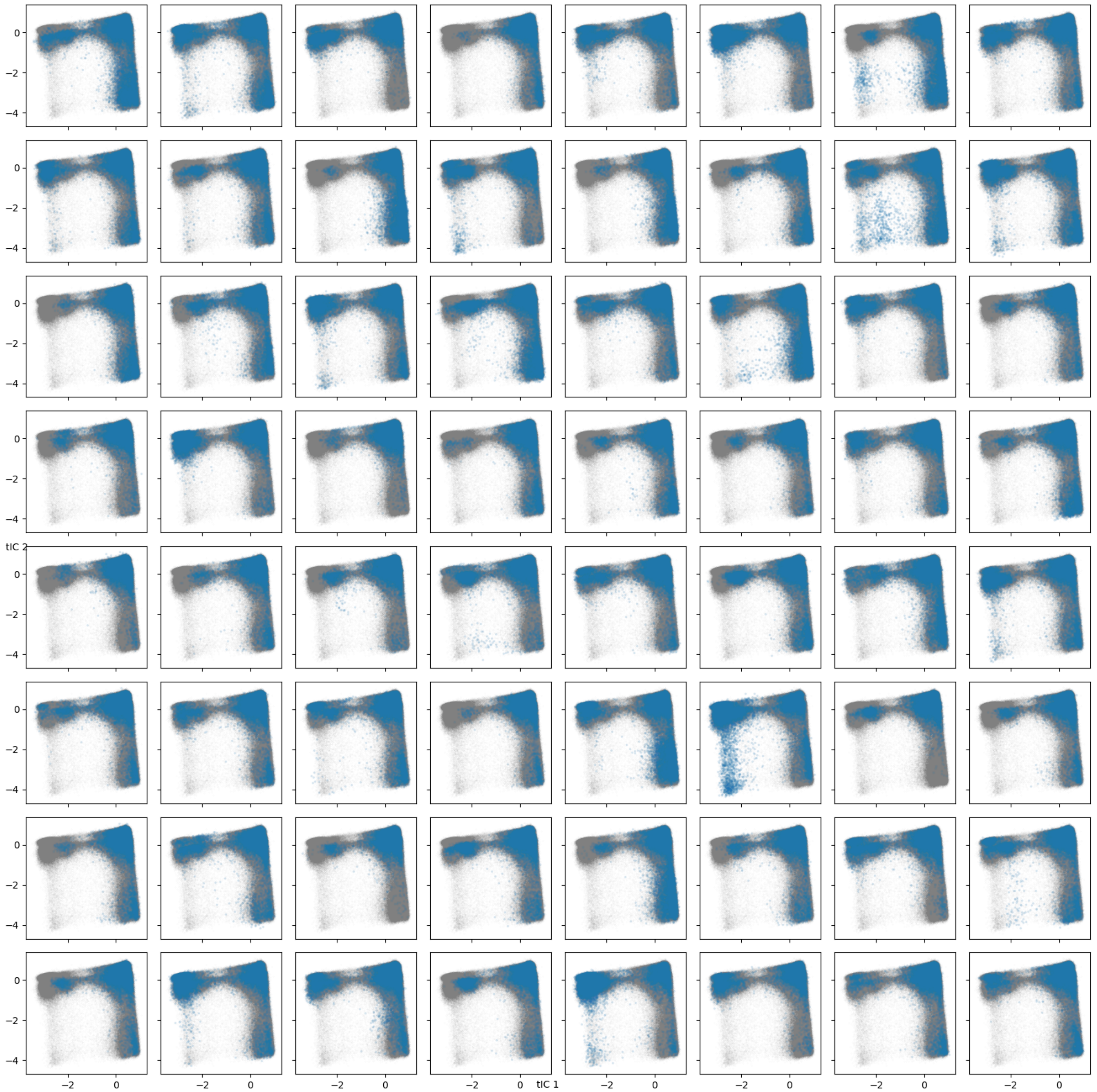

Figure S1: Time-lagged independent component analysis (tICA) of the human Pin1 WW-domain (PDB: 2f21). TICA was computed on 64 1 µs molecular dynamics (MD) trajectories, using a lag time $\tau = 0.25\,\mathrm{ns}$. Projections of all 64 trajectories onto the two leading tICs are shown in grey, projections of single trajectories are shown in blue.

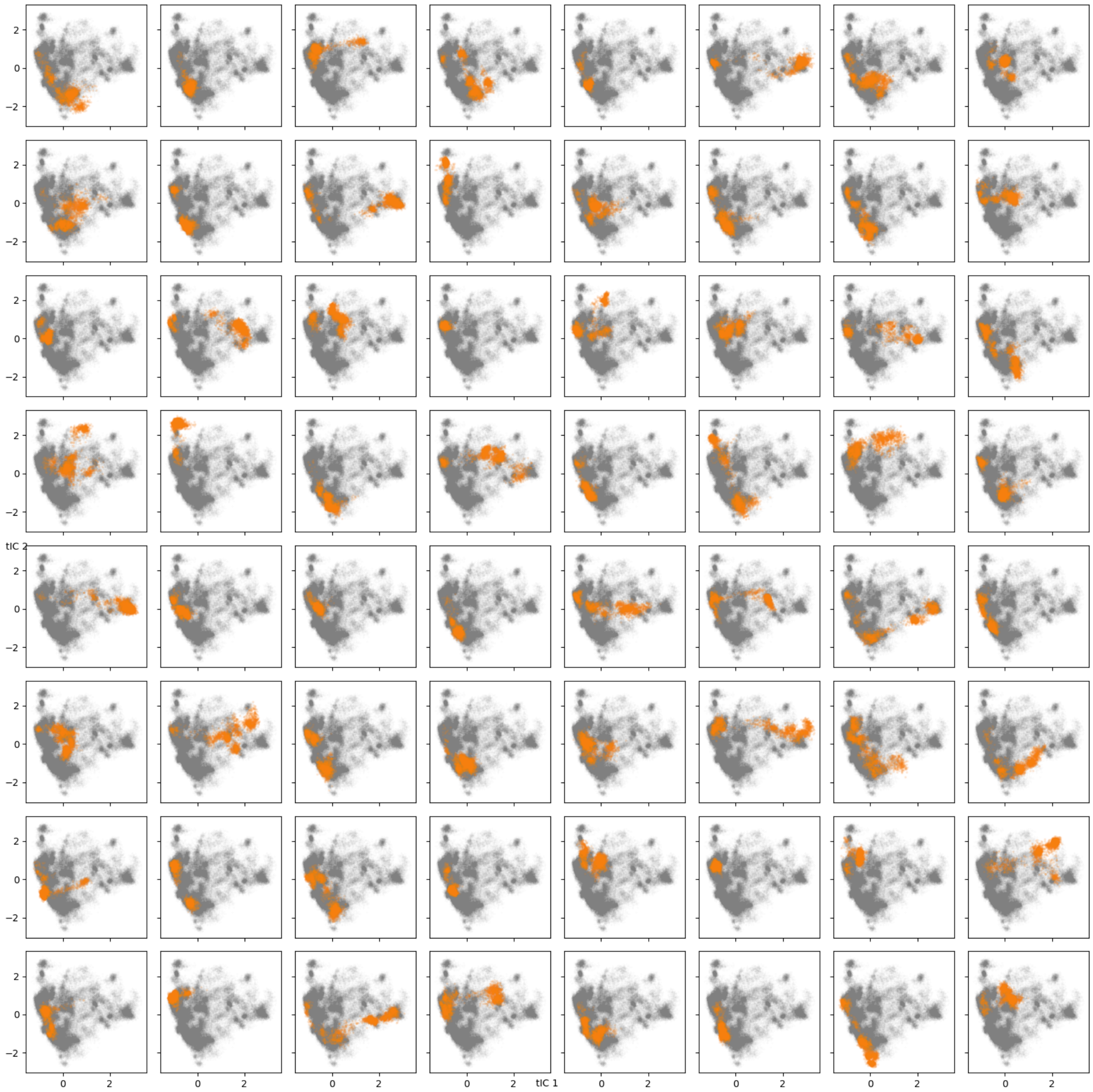

Figure S2: Time-lagged independent component analysis (tICA) of the homeodomain of mouse hepatocyte nuclear factor 6 (PDB: 1S7E). TICA was computed on 64 1 µs molecular dynamics (MD) trajectories, using a lag time $\tau = 2\,\text{ns}$. Projections of all 64 trajectories onto the two leading tICs are shown in grey, projections of single trajectories are shown in orange.

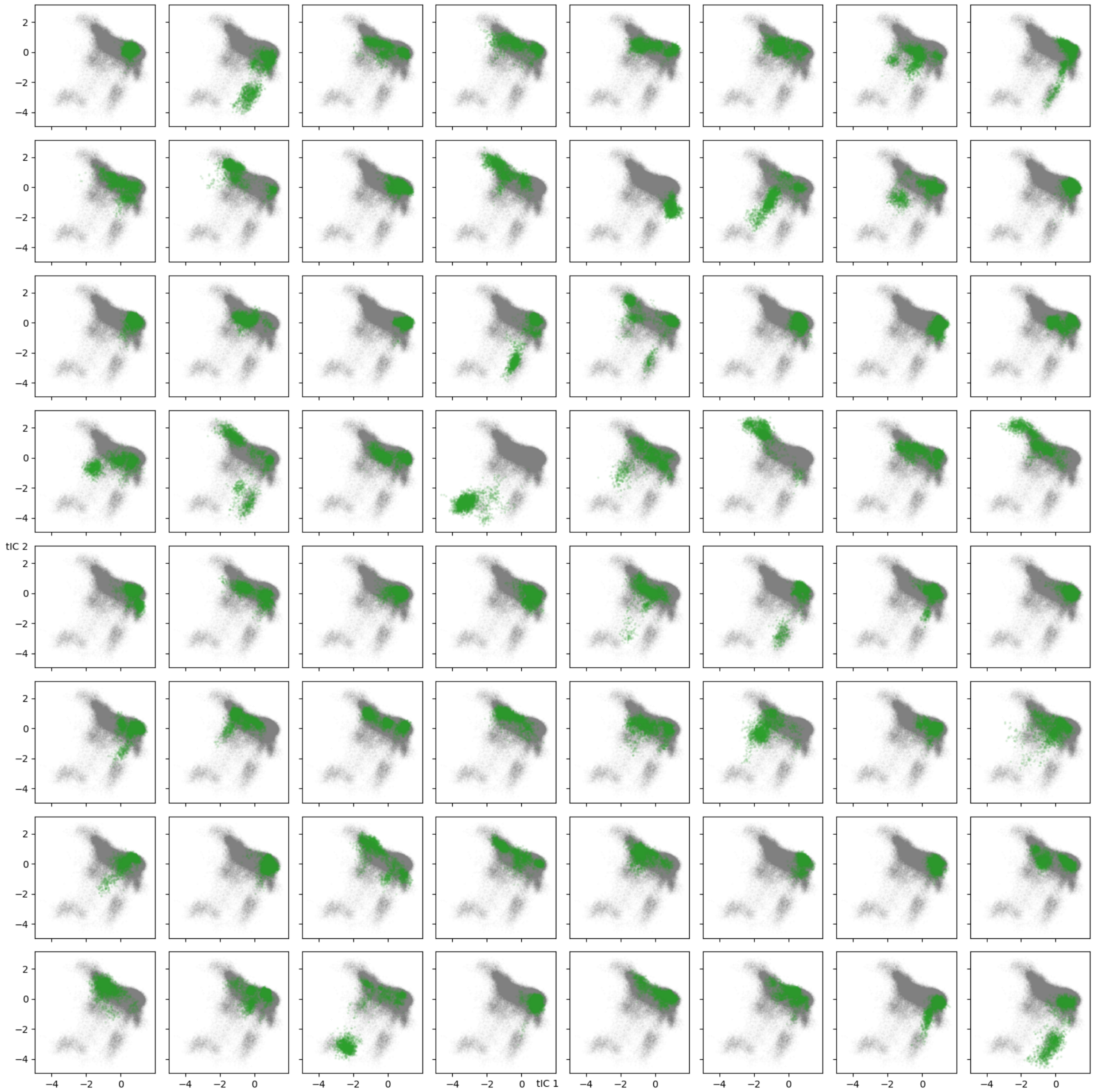

Figure S3: Time-lagged independent component analysis (tICA) of the XPC-binding domain of protein hHR23B (PDB: 1PVE). TICA was computed on 64 1 µs molecular dynamics (MD) trajectories, using a lag time $\tau = 2\,\text{ns}$. Projections of all 64 trajectories onto the two leading tICs are shown in grey, projections of single trajectories are shown in green.

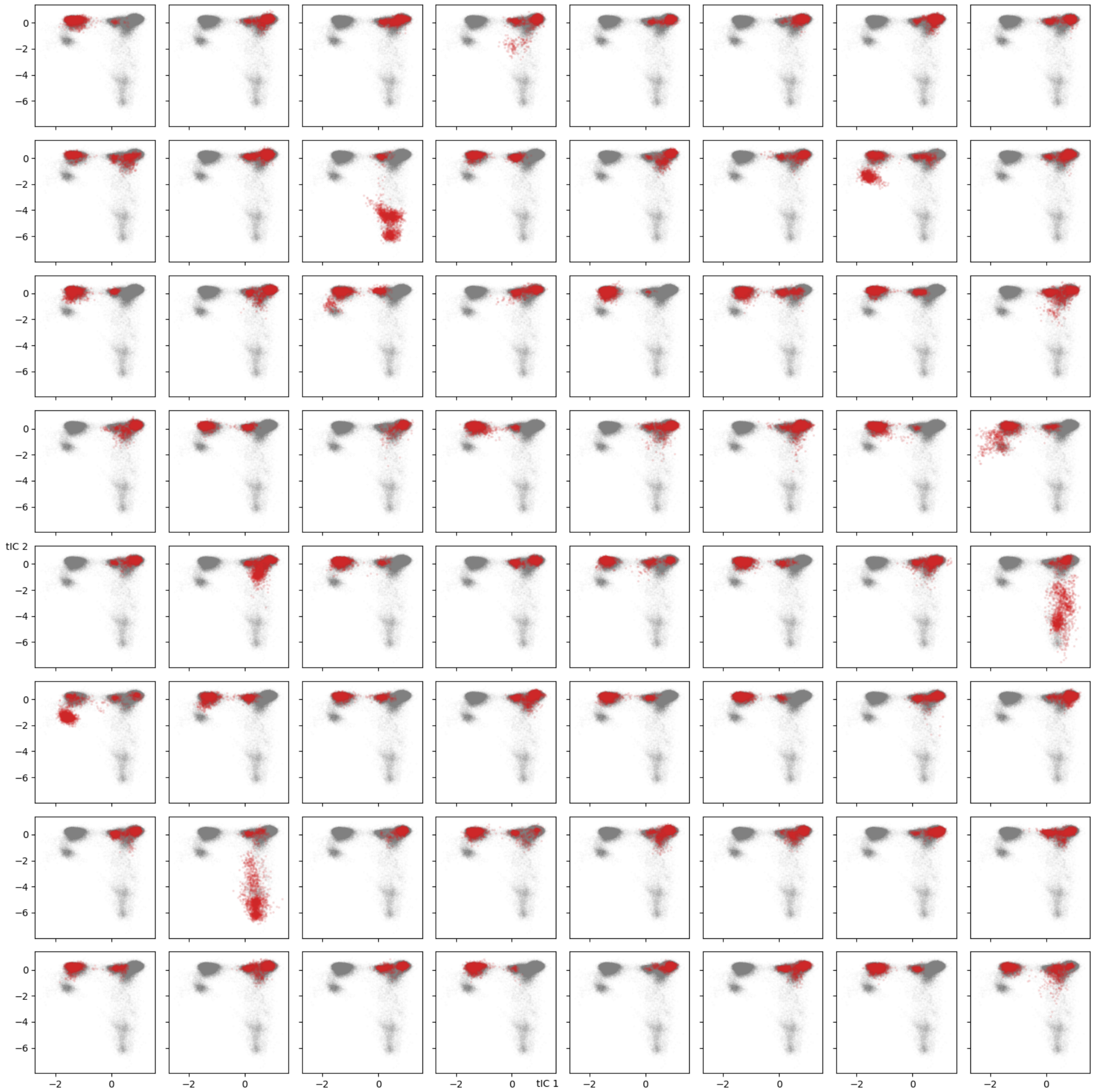

Figure S4: Time-lagged independent component analysis (tICA) of the protein neuronal nitric oxide synthase (PDB: 1QAU). TICA was computed on 64 1 µs molecular dynamics (MD) trajectories, using a lag time $\tau = 2\,\text{ns}$. Projections of all 64 trajectories onto the two leading tICs are shown in grey, projections of single trajectories are shown in red.
